# Selective Sugar Recognition by Anthracene-Type Boronic Acid Fluorophore/Cyclodextrin Supramolecular Complex Under Physiological pH Condition

**DOI:** 10.3389/fchem.2019.00806

**Published:** 2019-11-27

**Authors:** Ko Sugita, Yuji Tsuchido, Chisato Kasahara, Maria Antonietta Casulli, Shoji Fujiwara, Takeshi Hashimoto, Takashi Hayashita

**Affiliations:** ^1^Department of Materials and Life Sciences, Faculty of Science and Technology, Sophia University, Tokyo, Japan; ^2^Department of Life Science and Medical Bioscience, Graduate School of Advanced Science and Engineering, Waseda University (TWIns), Tokyo, Japan; ^3^Department of Current Legal Studies, Faculty of Law, Meiji Gakuin University, Yokohama, Japan

**Keywords:** cyclodextrin, boronic acid, supramolecular chemistry, fluorescence, sugar recognition

## Abstract

We synthesized novel PET (photoinduced electron transfer)-type fluorescence glucose probe **1** [(4-(anthracen-2-yl-carbamoyl)-3-fluorophenyl)boronic acid], which has a phenylboronic acid (PBA) moiety as the recognition site and anthracene as the fluorescent part. Although the PBA derivatives dissociate and bind with sugar in the basic condition, our new fluorescent probe can recognize sugars in the physiological pH by introducing an electron-withdrawing fluorine group into the PBA moiety. As a result, the p*K*_a_ value of this fluorescent probe was lowered and the probe was able to recognize sugars at the physiological pH of 7.4. The sensor was found to produce two types of fluorescent signals, monomer fluorescence and dimer fluorescence, by forming a supramolecular 2:1 complex of **1** with glucose inside a γ-cyclodextrin (γ-CyD) cavity. Selective ratiometric sensing of glucose by the **1**/γ-CyD complex was achieved in water at physiological pH.

## Introduction

Sugars, especially monosaccharides, play important roles in various biological systems (Brewer et al., 2002; Davis, 2002; Brodesser et al., 2003). There are many types of monosaccharides, such as glucose, galactose, fructose, etc., and their roles or abundance ratios *in vivo* are different. However, their molecular weights are identical, and each monosaccharide has various steric conformations in water. These facts have made their identification by molecular recognition difficult. Selective sugar recognition has captured the interest of disease, cancer, medical care, or food chemistry researchers (Ohtsubo and Marth, 2006; Jin et al., 2010; Xu et al., 2013). The conventional glucose sensor is an enzyme-type sensor that uses glucose oxidase (GOx) and enables the highly selective and quantitative analysis of glucose in the physiological condition (Sierra et al., 1997; White and Harmon, 2002; Endo et al., 2006; Kanekiyo and Tao, 2006; Ekanayake et al., 2007; Ye et al., 2010; Sun and James, 2015). However, the enzyme method is expensive and unstable due to denaturation. Therefore, the development of a stable non-enzyme-type chemical (artificial) glucose sensor is in great demand.

Over the past several decades, the development of sugar recognition molecules using phenylboronic acid (PBA) has been widely conducted (James et al., 1996; Kanekiyo and Hiraoki, 2005; Anslyn, 2007; Egawa et al., 2007, 2009; Fujita et al., 2008; Minami et al., 2014; Iwami et al., 2015; Tsuchido et al., 2015, 2016, 2017; Zhai et al., 2015; Shimomura et al., 2016; Wu et al., 2016, 2017; Nishiyabu et al., 2018). PBA is known to form cyclic boronate ester with the *cis*-diol group in the sugar. There are many reports of glucose sensors using PBA. Examples include colorimetric-type sensors that are based on absorption spectrum shift (Egawa et al., 2007, 2009), excimer fluorescence-type sensors (Kanekiyo and Hiraoki, 2005), micellar-type sensors that are based on hydrophobic interaction (Tsuchido et al., 2016; Wu et al., 2016), and near-infrared fluorescence-type sensors (JoSai-Red) (Shimomura et al., 2016).

Our research group has developed pyrene-type, anthracene-type, and naphthalene-type sugar recognition fluorescent PBA sensors (Ozawa et al., 2008; Kumai et al., 2012; Kano et al., 2014; Soma et al., 2018; Hashimoto et al., 2019), all of which are photoinduced electron transfer (PET)-type sensors. When these sensors bind with sugar, fluorescence emission by PET inhibition between the fused ring and the PBA moiety takes place. A unique phosphate anion sensor based on the macrocyclic boron complex at PBA-anthracene probe using PET mechanism was reported by Kameta and Hiratani (2006). However, anthracene and pyrene are highly hydrophobic and show poor solubility in water. Cyclodextrin (CyD) is an efficient host molecule that is able to incorporate various organic molecules in water. As a hydrophobic sensor (or probe) is included inside the CyD cavity, the formation of a stable supramolecular complex in water is expected.

This supramolecular inclusion system has several merits: (1) fluorescence emission can be enhanced by the formation of an inclusion complex of the fluorescent moiety and the hydrophobic CyD cavity; (2) selective glucose recognition can be expected by the 2:1 binding of PBA with glucose inside the CyD cavity; and (3) selective dimer fluorescence can be expected by the dimer formation of fluorophores, realizing ratiometric analysis. Generally, it is difficult to achieve glucose selectivity in a 1:1 binding system because PBA has a high binding constant for fructose compared to that for glucose. In contrast, the multi-point recognition-type sugar sensor having two (or more) PBA recognition sites is known to realize glucose selectivity by forming a 2:1 complex with glucose (James et al., 1994).

Recently, we have shown an efficient dimer formation for selective glucose sensing, in which two fluorescent molecules are included inside a CyD cavity and emits dimer fluorescence at long wavelengths (Hashimoto et al., 2019). However, this system realizes glucose recognition only in the basic condition (pH 11) due to the high p*K*_a_ value of PBA. Coordination of hydroxide to PBA to form tetrahedral boronate occurs only in the basic condition (>pH 9). Thus, the sugar recognition function is usually evaluated in the basic condition (>pH 9), as shown in previous reports using PBA sensors.

**Figure 1:**
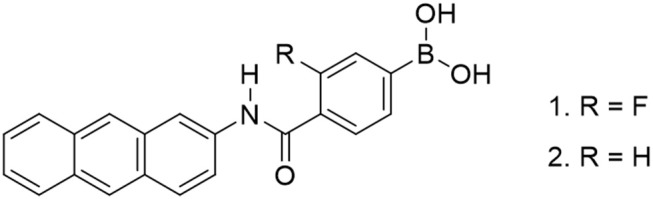
Chemical structure of anthracene boronic acid probes.

In this study, we introduced an electron-withdrawing fluorine group into the phenyl group of the PBA moiety to reduce the p*K*_a_ value of PBA (Figure 1). Several articles have reported the introduction of fluorine into PBA (Shoji and Freund, 2002; Deorea and Freund, 2003; Matsumoto et al., 2013), but there are no reports of supramolecular CyD-inclusion complex systems. Herein we report the introduction of fluorine into PBA to reduce the p*K*_a_ value of the probe, and selective glucose sensing at physiological pH in water, by measurements of fluorescence spectra and induced circular dichroism (ICD) spectra.

## Experimental Section

### Apparatus

Fluorescence spectra were measured with a Hitachi F-7000 fluorescence spectrometer equipped with a Peltier thermocontroller (Hitachi High-Tech Science Co., Tokyo, Japan) and a 1.0-cm quartz cell. Fluorescence emission from 350 to 600 nm was monitored at the excitation wavelength of 323 nm with a 2.5-nm slit width. UV-vis spectra were measured with JASCO V-570 and JASCO V-560 UV-vis spectrophotometers equipped with a Peltier thermocontroller (JASCO Co., Tokyo, Japan) and a 1.0-cm quartz cell. Circular dichroism (CD) spectra were recorded with a JASCO J-820 spectrophotometer equipped with a Peltier thermocontroller (JASCO Co., Tokyo, Japan) and a 1.0-cm quartz cell. ^1^H NMR spectra were obtained with a JEOL JNM-ECX 300 spectrometer or a JEOL JNM-ECA 500 spectrometer (JEOL Ltd., Tokyo, Japan) at 300 K. Elemental analysis was performed using a PerkinElmer 2400 Series II CHNS/O Elemental Analyzer (PerkinElmer, Inc., MA, USA). ESI-MS spectra were recorded on a JEOL JMS-T100LC instrument (JEOL Ltd., Tokyo, Japan). All synthetic reactions were monitored by thin-layer chromatography (TLC) on silica gel 60 F_254_ plates (Merck). All pH values were recorded with a Horiba F-52 pH meter (HORIBA Ltd., Kyoto, Japan).

### Reagent and Chemicals

4-Carboxy-3-fluorophenylboronic acid, pinacol, 4-(4,6-dimethoxy-1,3,5-triazin-2-yl)-4-methylmorpholinium chloride *n*-hydrate (DMT-MM), tetrahydrofuran, hydrochloric acid, sodium hydroxide, β-cyclodextrin, γ-cyclodextrin, fructose, glucose, galactose, phosphoric acid, sodium chloride, and dimethyl sulfoxide were purchased from FUJIFILM Wako Pure Chemical Corporation (Osaka, Japan). 1,4-Dioxane, chloroform, acetonitrile, and dimethyl sulfoxide-*d*_6_ were purchased from Kanto Chemical, Co., Inc. (Tokyo, Japan). 2-Aminoanthracene was purchased from Sigma-Aldrich Japan, Co., LLC (Tokyo, Japan). All organic solvents and reagents were commercially available with guaranteed grades and used without further purification. Water was doubly distilled and deionized using a Milli-Q water system (WG222, Yamato Scientific Co., Ltd., Tokyo, Japan, and Autopure WR-600G, Merck Millipore, MA, USA) before use.

### Synthesis of Probe 1

4-Carboxy-3-fluorophenylboronic acid (379 mg, 2.00 mmol) and pinacol (548 mg, 4.00 mmol) were dissolved in 1,4-dioxane (10 ml), and the reaction mixture was stirred for 24 h at room temperature. 1,4-Dioxane was removed *in vacuo* and the residue was dissolved in a small amount of chloroform. The sample was purified by size exclusion chromatography and dried *in vacuo* to give a white solid (**FPBAp**). **FPBAp** (372 mg, 1.00 mmol), 2-aminoanthracene (194 mg, 1.00 mmol), and DMT-MM (430 mg, 1.50 mmol) were dissolved in tetrahydrofuran (20 ml) and refluxed for 24 h. The solvent was removed *in vacuo*. The residue was purified by extraction (chloroform/water) and size exclusion chromatography. The obtained product was dissolved in acetonitrile–water (adjusted to pH 3 by using hydrochloric acid) mixture (1:1 v/v, 60 ml) and the solution was stirred for 36 h at room temperature to remove the pinacol protection. The solution was filtered and the residue was washed with water and dried *in vacuo* to give a yellow-brown solid (162 mg, 0.418 mmol, 45%). The obtained product was analyzed by ^1^H NMR measurement, elemental analysis, and negative ESI-HRMS. ^1^H NMR (Figure S1, 300 MHz, DMSO-*d*_6_); δ (ppm): 10.65 (s, 1H), 8.63 (s, 1H), 8.49 (d, 2H), 8.40 (s, 2H), 8.05 (t, 3H), 7.62–7.75 (m, 4H), 7.41–7.53 (m, 2H); Anal. calcd for C_21_H_15_BFNO_3_ (%): C, 70.23; H, 4.21; N, 3.90; C/N, 18.0. Found: C, 70.35; H, 4.40; N, 3.95; C/N, 17.8; negative ESI-HRMS (Figure S2): *m*/*z* calcd. for [M−3H+2CH_3_]^−^: 386.1364, Found: 386.1374. This peak means deprotonation (−1H) and methoxylation (−2OH + 2OCH_3_), which was caused by ESI-MS-ionization step in methanol solvent.

### Evaluation of Formation of Boronic Acid Probe/CyD Supramolecular Complex

To evaluate the formation of boronic acid probe/CyD supramolecular complex, UV-vis and fluorescence spectral measurements were performed. UV-vis and fluorescence spectra were measured in various pH conditions in the absence or presence of sugar (30 mM).

### Evaluation of Electron-Withdrawing Effect of Boronic Acid Probe on Sugar Recognition Function

To evaluate the fluorescence response of the **1**/γ-CyD complexes toward various sugars, fluorescence spectral measurements were performed. Solutions containing **1** (10 μM), CyD (5 mM), sugar (0 or 30 mM), NaCl (100 mM), and phosphate buffer (10 mM, adjusted to pH 7.4) were prepared and fluorescence spectra were measured at pH 7.4. The solutions were also observed visually using UV-vis handy light.

### Evaluation of CD Response of Boronic Acid Probe/CyD Supramolecular Complex

Solutions containing probe **1** (10 μM), γ-CyD (5 mM), sugar (0–30 mM), phosphate buffer (10 mM, adjusted to pH 7.4), and NaCl (100 mM) were prepared. To investigate the supramolecular complex formation of **1** and γ-CyD, CD spectra at pH 7.4 were measured.

### Sugar Titration Measurements of 1/γ-CyD Complexes

To evaluate the sugar response of the **1/**γ-CyD complexes, fluorescence spectral measurements were performed. Solutions containing probe **1** (10 μM), γ-CyD (5 mM), sugar (0–30 mM), phosphate buffer (10 mM, adjusted to pH 7.4), and NaCl (100 mM) were prepared and fluorescence spectra were measured at pH 7.4 with the addition of sugar.

## Results and Discussion

### Evaluation of Formation of Boronic Acid Probe/CyD Supramolecular Complex

**Figure 2:**
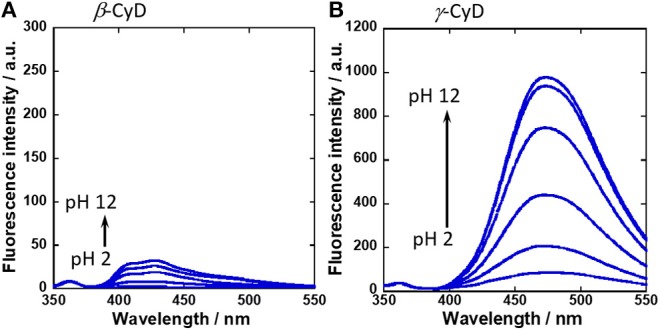
Fluorescence spectral changes of **(A) 1**/β-CyD complex with glucose and **(B) 1**/γ-CyD complex with glucose in 2% DMSO−98% water (v/v) with various pH. [**1**] = 10 μM, [CyD] = 5 mM, [glucose] = 30 mM, [NaCl] = 100 mM, [phosphate buffer] = 10 mM, λ_ex_ = 323 nm.

**Figure 3:**
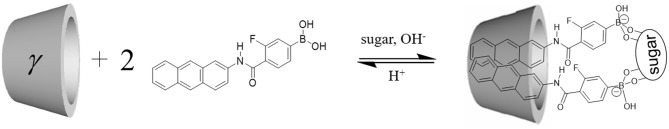
Estimated structure of **1**/γ-CyD complex.

To evaluate the function of fluorescent probe **1**, the binding of **1** with sugars was confirmed by ^19^F NMR spectra (Figure S3). Then, UV-vis absorption spectra were measured by changing the solution pH (Figure S4). From pH profile measurements, the isosbestic point was confirmed at 323 nm. Thus, the excitation wavelength for fluorescence measurement was set at 323 nm. Figure 2 shows the fluorescence spectra of **1** in the presence of β-CyD or γ-CyD with various pH. The fluorescence maximum of **1** in the presence of β-CyD was 432 nm and that of **1** in the presence of γ-CyD was 472 nm. This difference in fluorescence maximum was derived from the difference in CyD cavity size (Ueno et al., 1980; Ke et al., 2009; Hashimoto et al., 2019). It is well-known that CyDs are cyclic oligosaccharides composed of glucose units (β-CyD has seven glucose units and γ-CyD has eight units). Because β-CyD has a small cavity, only one molecule of **1** could be included in its cavity. Thus, the **1**/β-CyD complexes emitted monomer fluorescence. On the other hand, γ-CyD has a larger cavity than β-CyD, and so probe **1** and γ-CyD formed 2:1 complexes and emitted dimer fluorescence (Figure 3). We had previously reported that probe **2** showed similar results; **2**/β-CyD complexes emitted monomer fluorescence and **2**/γ-CyD complexes emitted dimer fluorescence (Hashimoto et al., 2019). This result indicated that the formation of a supramolecular complex was also achieved even if the probe possessed a fluorine moiety.

### Evaluation of Electron-Withdrawing Effect of Boronic Acid Probe on Sugar Recognition Function

**Figure 4:**
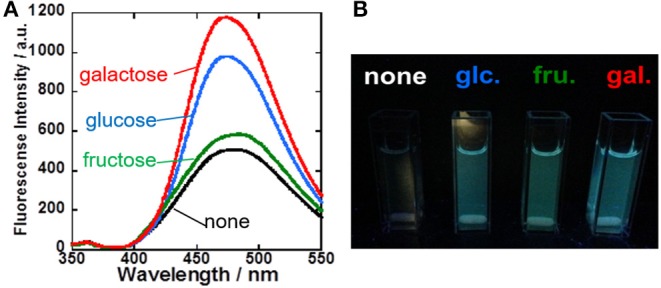
**(A)** Fluorescence spectra of **1**/γ-CyD complex with various sugars (none: black; green: fructose; blue: glucose; red: galactose) in 2% DMSO−98% water (v/v) at basic condition around pH 10. [**1**] = 10 μM, [γ-CyD] = 5 mM, [sugar] = 0 or 30 mM, [phosphate buffer] = 10 mM, [NaCl] = 100 mM, λ_ex_ = 323 nm. **(B)** Fluorescence image of **1**/γ-CyD complex solution without and with 30 mM of various sugars at pH 7.4 under UV irradiation.

To evaluate the sugar recognition function of the **1**/γ-CyD complexes, the fluorescence spectra of the **1**/γ-CyD complexes in the presence of various sugars were carried out. Fluorescence intensity of the **1**/γ-CyD complexes was gradually increased by addition of various sugars at basic condition (Figure 4A), similar behavior as **2**/γ-CyD complexes. Moreover, **1**/γ-CyD complexes emitted fluorescence by recognizing various sugars even at physiological pH condition. Figure 4B shows an optical observation image of the **1**/γ-CyD complexes in the presence of various sugars at pH 7.4. The fluorescence by sugar recognition could be observed by the naked eye. The pH profiles of the **1**/γ-CyD complexes with other sugars were measured (Figure S5). Dimer fluorescence at 472 nm increased in the basic condition. The apparent p*K*_a_ values of the **1**/γ-CyD complexes with or without sugars were calculated by curve fitting. The p*K*_a_ values of the **1**/γ-CyD complexes without sugar and with glucose, fructose, and galactose were 8.57 (±0.05), 7.78 (±0.03), 7.22 (±0.14), and 7.63 (±0.05), respectively. To discuss the effect of the fluorine moiety of the boronic acid probe, the p*K*_a_ values of the probe were compared. The p*K*_a_ values of non-fluorinated anthracene-type boronic acid probe **2** (Hashimoto et al., 2019) without sugar, with glucose and with fructose were 10.94 (±0.13), 10.85 (±0.03), and 9.92 (±0.09), respectively (Figure S6). Compared with probe **2**, probe **1** has much lower p*K*_a_ values, indicating the electron-withdrawing effect of the fluorine group of the boronic acid probes. The introduction of the fluorine moiety into the probe realized sugar recognition in the neutral pH condition.

**Figure 5:**
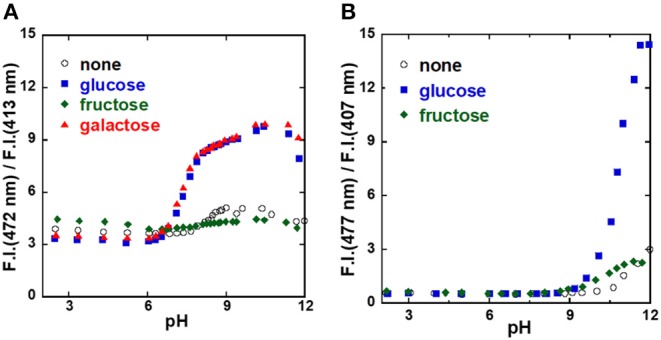
pH profiles of probe [**(A) 1**, **(B) 2**]/γ-CyD complexes in 2% DMSO−98% water (v/v). [probe] = 10 μM, [γ-CyD] = 5 mM, [sugar] = 0 or 30 mM, [phosphate buffer] = 10 mM, [NaCl] = 100 mM, λ_ex_ = 323 nm.

To investigate the sugar recognition ability of the **1**/γ-CyD complexes, the ratiometric fluorescence behavior was examined (Figure 5). Figure 5 shows the pH dependence of the ratio of dimer fluorescence intensity at 472 nm to monomer fluorescence at 413 nm. The ratio increased with glucose and galactose at physiological pH, and there was almost no change in the absence of sugar or in the presence of fructose. These results indicated that the **1**/γ-CyD complex formed a dimer inside the γ-CyD cavity in the presence of glucose or galactose, but not in the absence of sugar or in the presence of fructose. Thus, it is evident that the formation of the 2:1 complex of boronic acids with glucose or galactose took place inside the γ-CyD cavity. The sugar recognition ability of the **2**/γ-CyD complex was investigated as well. The dimer formation of **2**/γ-CyD with glucose was only noted at pH 11.5.

### Sugar Titration Measurements of 1/γ-CyD Complexes

**Figure 6:**
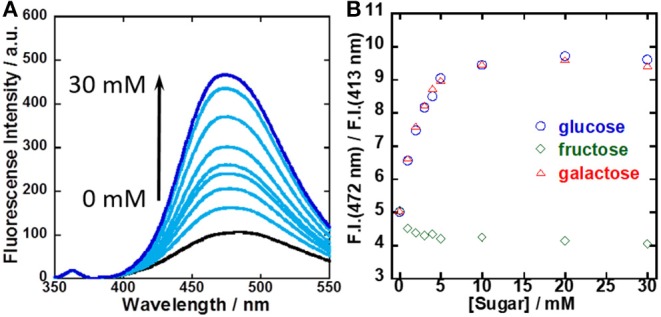
**(A)** Fluorescence spectral changes of **1**/γ-CyD complex in 2% DMSO−98% water (v/v) at pH 7.4 with the addition of glucose. **(B)** Ratiometric plots of **1**/γ-CyD complex in 2% DMSO−98% water (v/v) at pH 7.4 with the addition of various concentrations of sugars. [**1**] = 10 μM, [γ-CyD] = 5 mM, [sugar] = 0–30 mM, [phosphate buffer] = 10 mM, [NaCl] = 100 mM, λ_ex_ = 323 nm.

To evaluate the sensitivity for sugar recognition, UV-vis and fluorescence spectral changes of the **1**/γ-CyD complex at pH 7.4 with sugar concentration were evaluated (Figures S7, S8 and Figure 6A). It was evident that dimer fluorescence increased with the addition of glucose. Figure 6B shows the dependence of the ratio of dimer fluorescence intensity at 472 nm to monomer fluorescence intensity at 413 nm on sugar concentration. The dimer/monomer ratio increased with the addition of glucose and galactose and saturated at 10 mM. In contrast, the dimer/monomer ratio remained almost constant with the addition of fructose. From the results, the binding constants of **1**/γ-CyD to glucose and galactose were calculated as literature (Grynkiewicz et al., 1985; Shimpuku et al., 2009) and decided to be 1.44 × 10^7^ and 1.55 × 10^7^ M^−2^, respectively (see **Supplementary Section Calculation of the Binding Constant of 1/γ-CyD Complex With Sugar From Ratiometric Plots of Fluorescence Spectra** and Figure S9).

### Evaluation of CD Response of Boronic Acid Probe/CyD Supramolecular Complex

**Figure 7:**
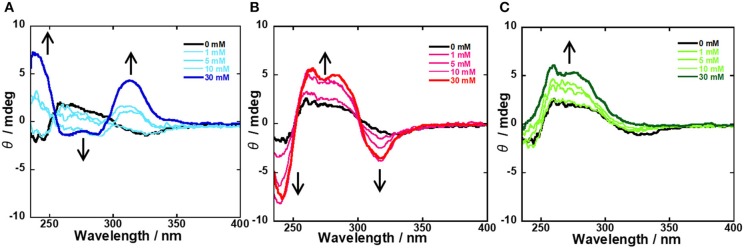
ICD spectral changes of **1**/γ-CyD complex in 2% DMSO−98% water (v/v) with the addition of various concentrations of sugars [**(A)** glucose, **(B)** galactose, **(C)** fructose] at pH 7.4. [**1**] = 10 μM, [γ-CyD] = 5 mM, [sugar] = 0–30 mM, [phosphate buffer] = 10 mM, [NaCl] = 100 mM.

To evaluate the supramolecular complexation of probe **1** and CyD, the ICD spectra of **1**/β-CyD and **1**/γ-CyD complexes at pH 7.4 were examined. When the boronic acid probe (guest molecule) is included in the CyD cavity, ICD and the Cotton effect are observed in the ICD spectrum. Figure S10 and Figure 7 shows the ICD spectra of the **1**/β-CyD and **1**/γ-CyD complexes with glucose, galactose, and fructose, respectively. For the **1**/β-CyD complexes, weak Cotton effect due to the complexation of CyD and anthracene was observed (the positive Cotton effect at 200–230 nm with fructose are caused by the absorption of fructose itself). On the other hand, in the case of the **1**/γ-CyD complex without sugar, a positive Cotton effect was observed in the shorter wavelength and a negative Cotton effect was observed in the longer wavelength. The observation of the Cotton effect indicated that probe **1** formed an inclusion complex with γ-CyD. This split-type Cotton effect denoted that two probes **1** existed as a twisted structure inside the γ-CyD cavity. In the presence of glucose (blue lines), the ICD spectra of the **1**/γ-CyD complex showed reverse splitting patterns relative to the ICD spectrum of the complex without sugar (black line). However, in the presence of galactose (red lines), the ICD spectra of the **1**/γ-CyD complex showed large splitting patterns. The ICD spectra of the **1**/γ-CyD complex with glucose and galactose showed strong splitting Cotton effects with the addition of sugar. The increase of the splitting indicated that complex formation of two boronic acid and one sugar molecule (glucose and galactose) fixed the twisted structure. The difference in the splitting patterns of the **1**/γ-CyD complexes with glucose and galactose demonstrated the difference of the twisting rotation directions of the **1**/γ-CyD-sugar complex. In the presence of fructose (green lines), the positive Cotton effect in the shorter wavelength was increased and the negative Cotton effect in the longer wavelength disappeared with the addition of fructose. This result indicated that the formation of the twisted structure was suppressed by fructose binding. Thus, we could discriminate glucose and galactose by the splitting patterns of the ICD spectra of the **1**/γ-CyD-sugar complexes. As a result, selective sugar recognition by ICD spectral analysis was achieved at physiological pH in 98% water−2% DMSO solution.

## Conclusion

We have developed a novel anthracene-type boronic acid probe possessing a fluorine moiety. By introducing the fluorine moiety into the PBA moiety, the p*K*_a_ value of the boronic acid probe could be reduced from 10 to 7. Thus, the boronic acid probe could efficiently recognize sugars at the physiological pH of 7.4 due to the electron-withdrawing effect of the fluorine group in the PBA moiety. Moreover, the ICD spectral response of the probe/γ-CyD complexes to various sugars differed markedly, thereby resulting in the selective discrimination of sugars based on the splitting pattern of the Cotton effect. This fluorinated boronic acid probe is highly promising as a selective sugar recognition sensor in the neutral pH condition.

## Data Availability Statement

All datasets generated for this study are included in the article/Supplementary Material.

## Author Contributions

KS, CK, and MC performed the experiments. KS, YT, THas, and THay wrote the manuscript. All authors designed the experiments and were involved in the data analysis. All authors designed the experiments, were involved in the data analysis, and have expressed approval of the final version of the manuscript.

### Conflict of Interest

The authors declare that the research was conducted in the absence of any commercial or financial relationships that could be construed as a potential conflict of interest.
