# Supplementary material for: Selective Sugar Recognition by Anthracene-Type Boronic Acid Fluorophore/Cyclodextrin Supramolecular Complex Under Physiological pH Condition
Source: Front Chem. 2019 Nov 27;7:806. doi: 10.3389/fchem.2019.00806 (PMC6890849; doi:10.3389/fchem.2019.00806)
Supplement: Supplementary file 1 [file Data_Sheet_1.pdf]

Supplementary Material

Contents

1. Characterization of the probe **1**

2. ^{19}F NMR spectra of **1** with/without sugar

3. UV-Vis and fluorescence spectra of **1**(or**2**)/*γ*-CyD complexes in various pH conditions

4. UV-Vis and fluorescence spectra of **1**/*γ*-CyD complexes in various sugar concentrations

5. Calculation of the binding constant of **1**/*γ*-CyD complex with sugar from ratiometric plots of fluorescence spectra

6. ICD spectra of **1**/*β*-CyD complex with addition of various concentration of sugars

1. Characterization of the probe 1

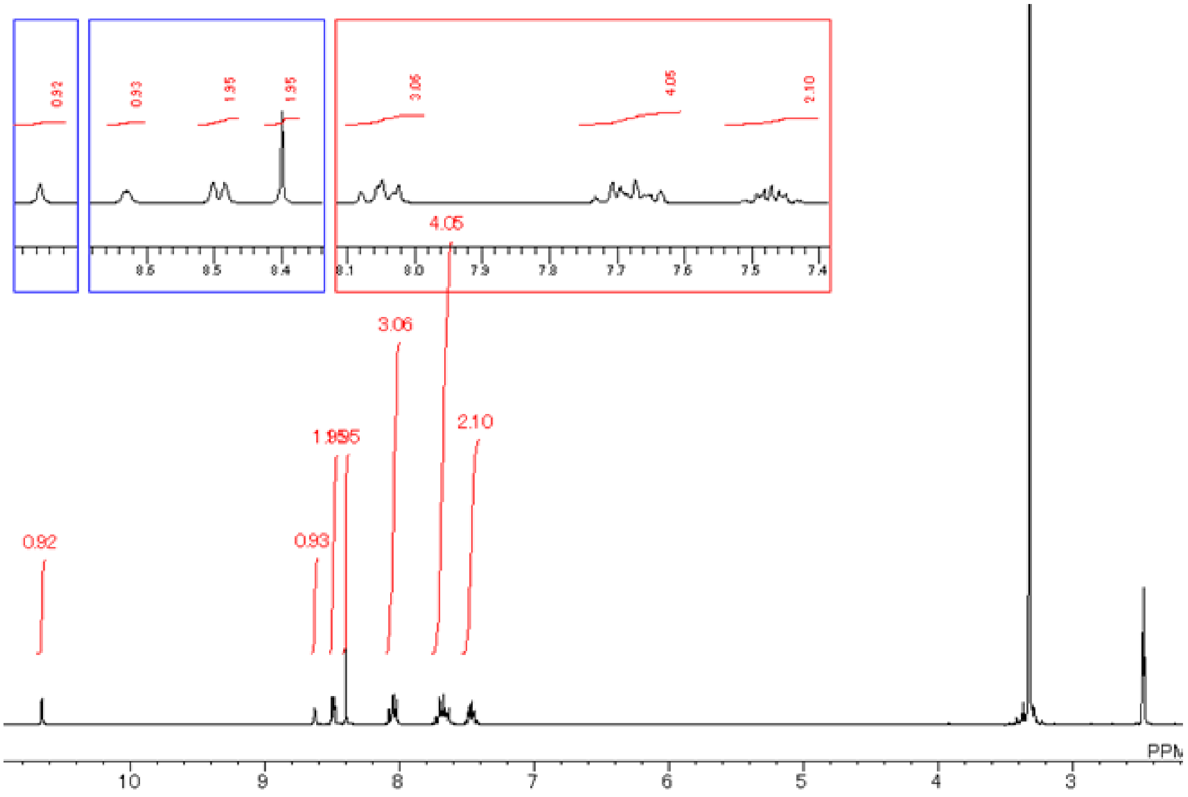

Figure S1. ^{1}H NMR spectra of the probe **1**. (300 MHz, DMSO-d_6)

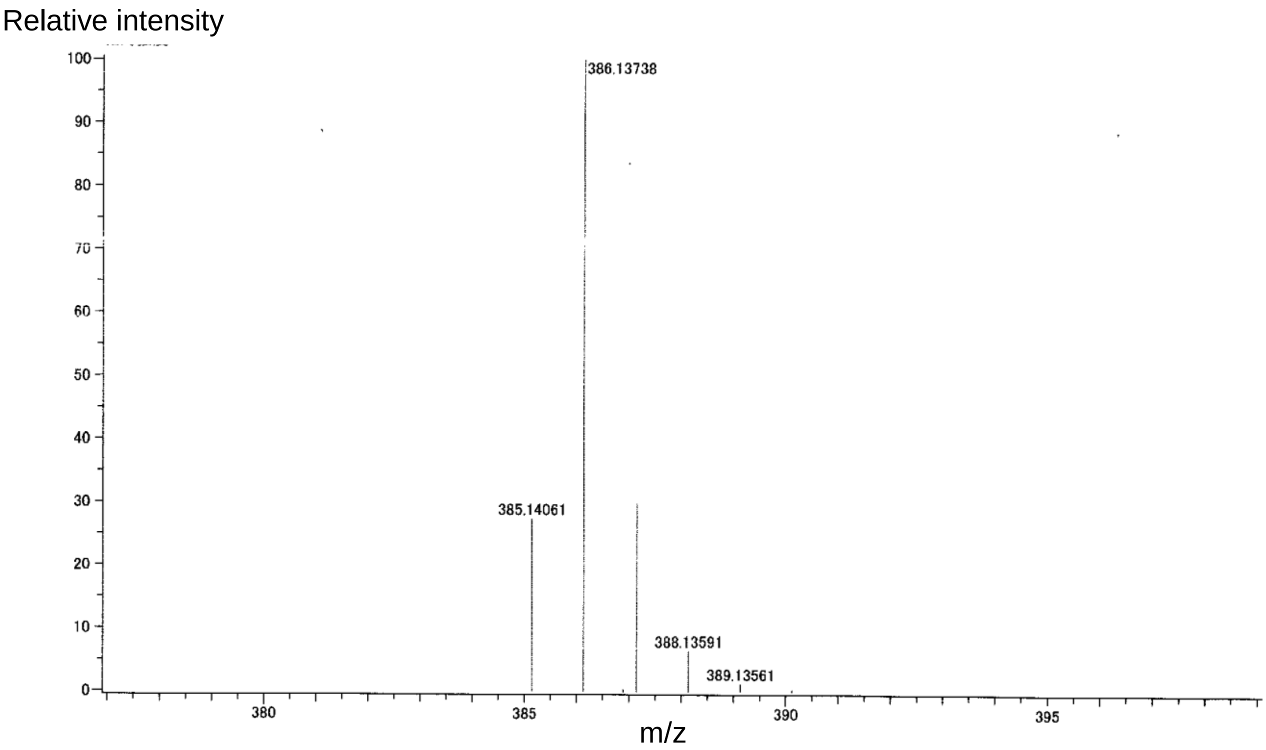

Figure S2. Negative ESI-HRMS of the probe **1**.

2. ^{19}F NMR spectra of 1 with/without sugar

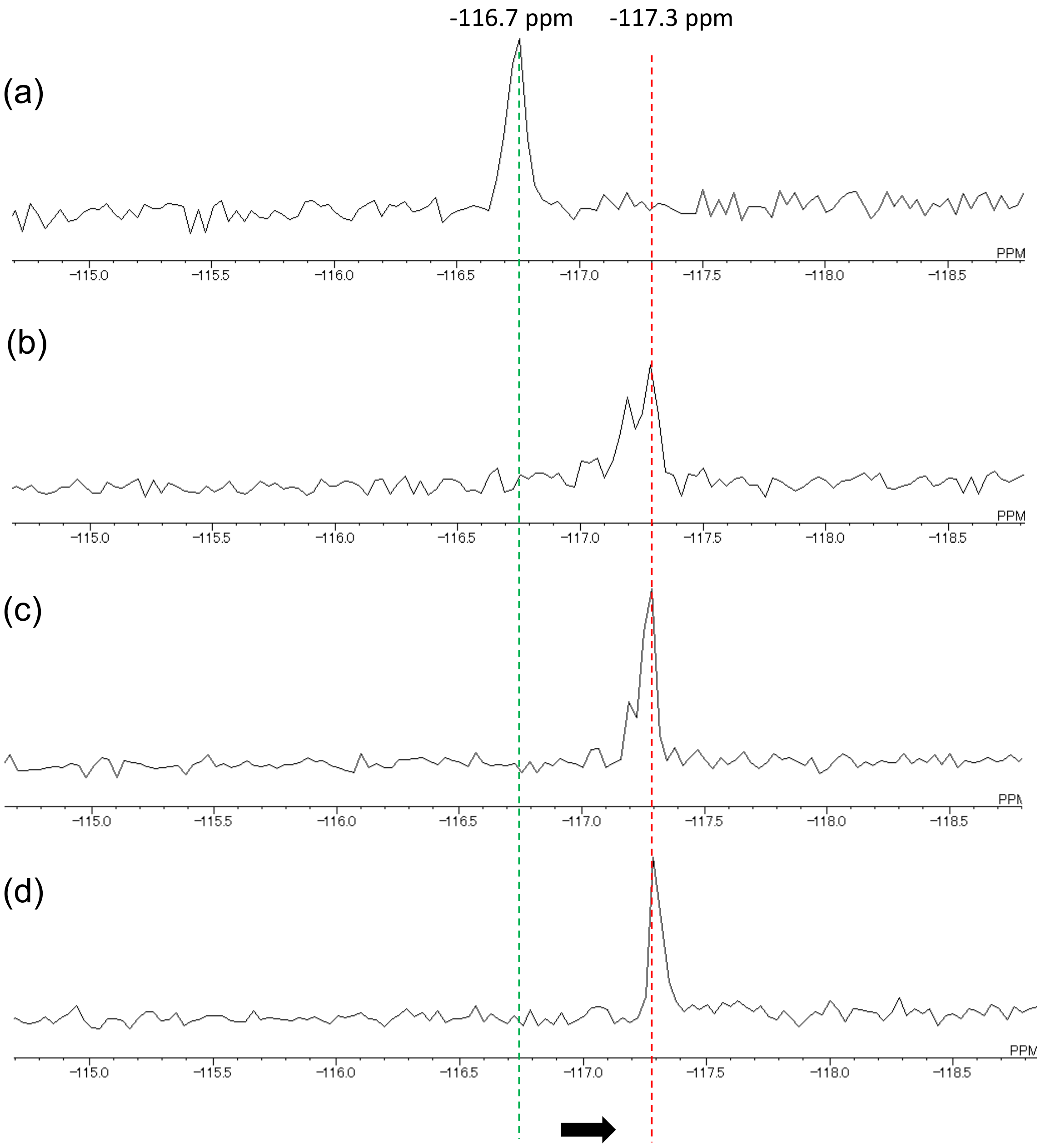

Figure S3. ^{19}F NMR spectra of **1** in 50% DMSO-d_6 - 50% D_2O (pD 10.0 carbonate buffer) (v/v) (a) without sugar, (b) glucose, (c) fructose, (d) galactose. [**1**] = 0.5 mM, [sugar] = 0 or 30 mM, [carbonate buffer] = 100 mM, [NaCl] = 100 mM, Number of scan 256.

3. UV-Vis and fluorescence spectra of 1(or 2)/γ-CyD complexes in various pH conditions

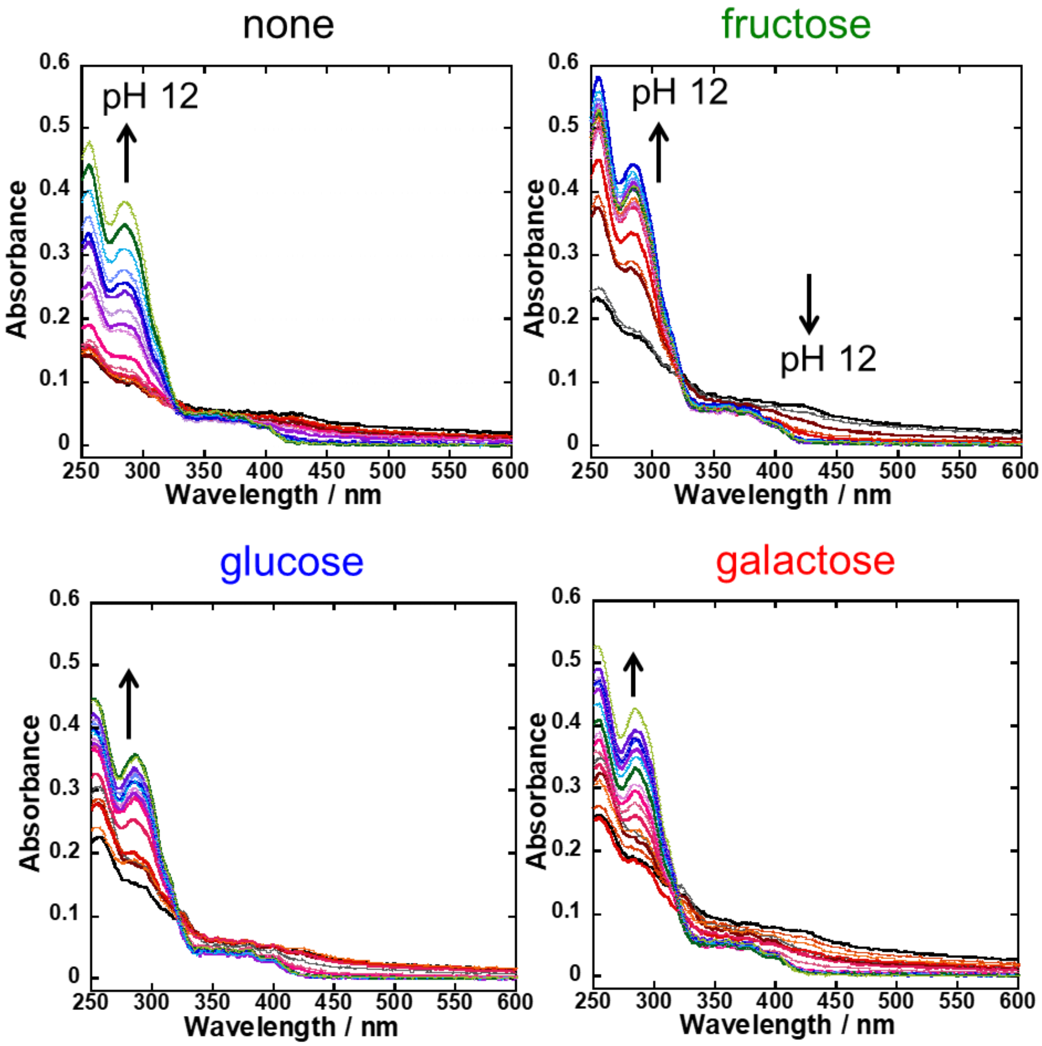

Figure S4. UV-Vis spectra of **1**/γ-CyD complexes with various sugars in 2% DMSO-98% water (v/v). [**1**] = 10 μM, [γ-CyD] = 5 mM, [sugar] = 0 or 30 mM, [phosphate buffer] = 10 mM, [NaCl] = 100 mM.

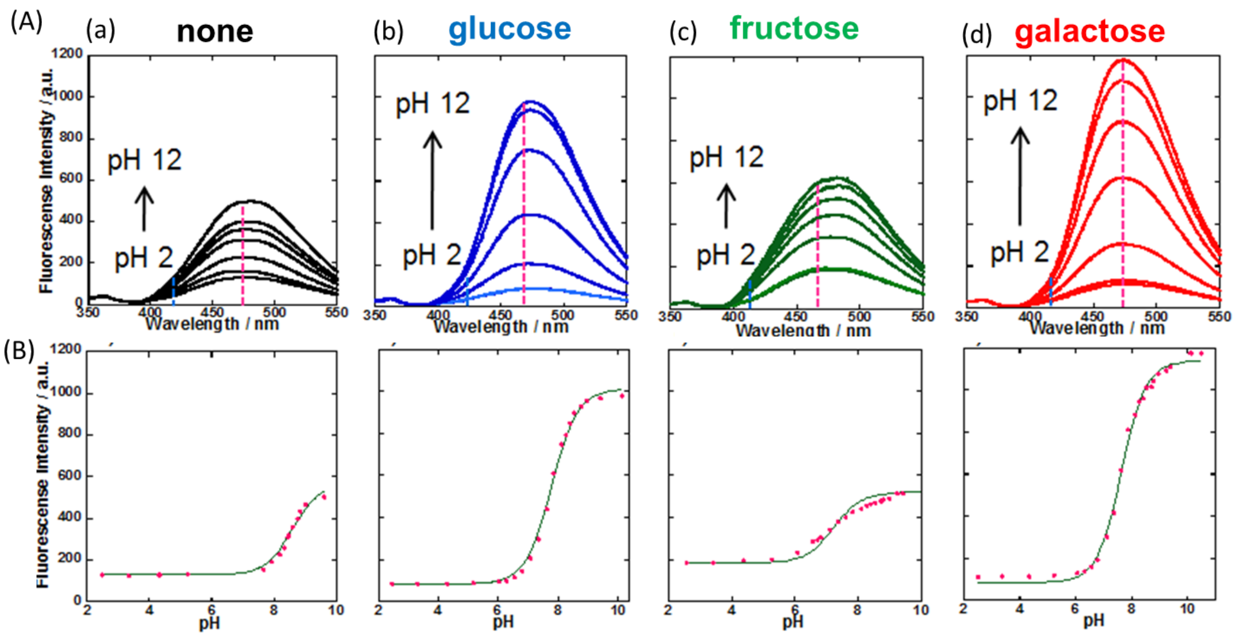

Figure S5. (A) Fluorescence spectra (B) Fluorescent maximum intensity at 472 nm of **1**/γ-CyD complexes in 2% DMSO-98% water (v/v) (a) without sugar, (b) with glucose, (c) with fructose, (d) with galactose. [**1**] = 10 μM, [γ-CyD] = 5 mM, [sugar] = 0 or 30 mM, [phosphate buffer] = 10 mM, [NaCl] = 100 mM. λ_{ex} = 323 nm.

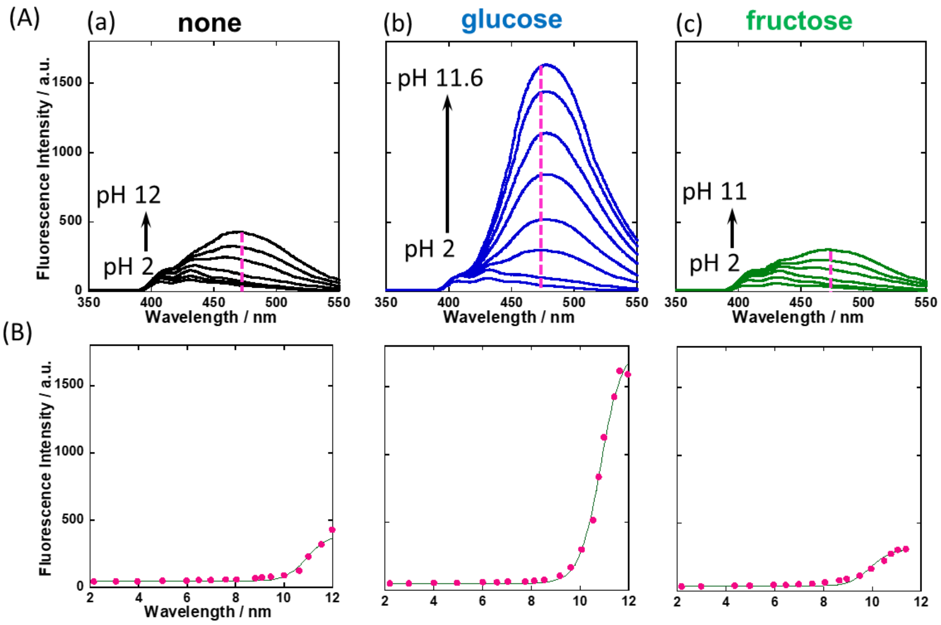

Figure S6. (A) Fluorescence spectra (B) Fluorescent maximum intensity at 472 nm of **2**/γ-CyD complexes in 2% DMSO-98% water (v/v) (a) without sugar, (b) with glucose, (c) with fructose. [**2**] = 5 μM, [γ-CyD] = 5 mM, [sugar] = 0 or 30 mM, [phosphate buffer] = 10 mM, [NaCl] = 100 mM. λ_{ex} = 300 nm.

4. UV-Vis and fluorescence spectra of 1/γ-CyD complexes in various sugar concentrations

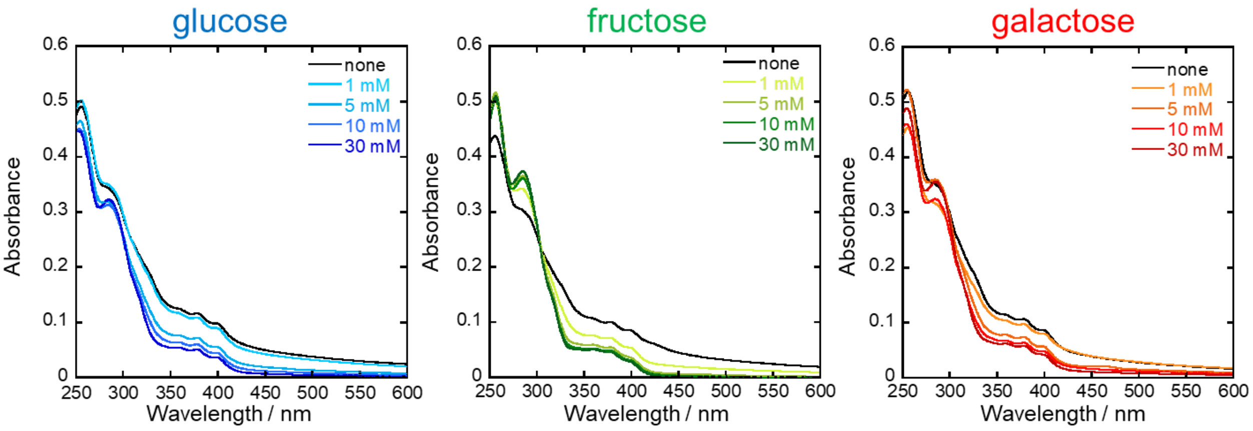

Figure S7. UV-vis spectra of **1**/γ-CyD complexes in 2% DMSO-98% water (v/v) by changing sugar concentration at pH 7.4. [**1**] = 10 μM, [γ-CyD] = 5 mM, [sugar] = 0-30 mM, [phosphate buffer] = 10 mM, [NaCl] = 100 mM. λ_{ex} = 323 nm.

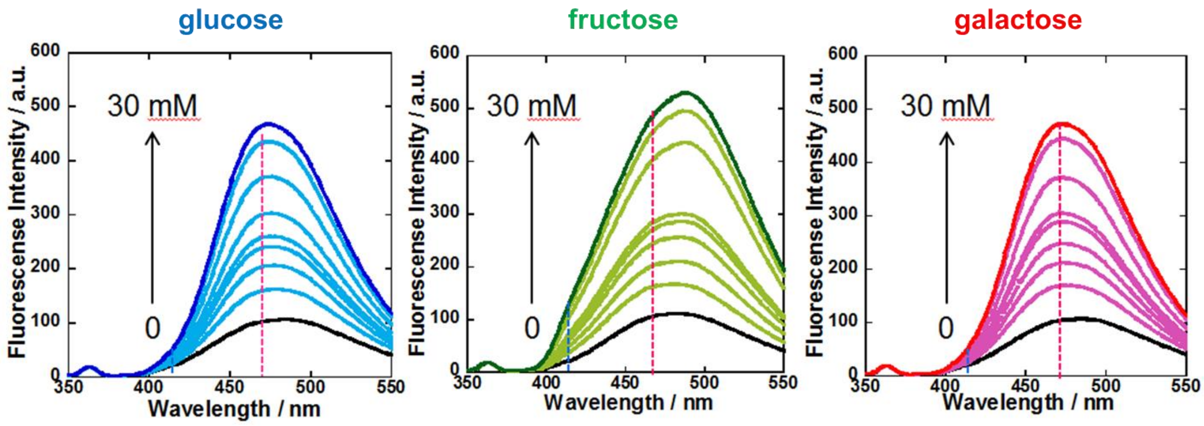

Figure S8. Fluorescence spectra of **1**/γ-CyD complexes in 2% DMSO-98% water (v/v) by changing sugar concentration at pH 7.4. [**1**] = 10 μM, [γ-CyD] = 5 mM, [sugar] = 0-30 mM, [phosphate buffer] = 10 mM, [NaCl] = 100 mM. λ_{ex} = 323 nm.

5. Calculation of the binding constant of 1/γ-CyD complex with sugar from ratiometric plots of fluorescence spectra

The apparent 2:1 binding constant (K_{21}) of **1** with sugars (glucose, galactose) in the presence of 5.0 mM γ-CyD was determined from the changes in the fluorescence spectra. With an increase in sugar concentration, the fluorescence intensity ratio (FI_{472}/FI_{413}) for **1** increased (Figure 5). On the assumption that the fluorescence change is only induced by the formation of a 2:1complex (L_2S) between **1** (L) and sugar (S), the binding constant K_{21} can be defined as follows:

$$2L + S \overset{K_{21}}{\rightleftharpoons} L_2S$$

$$K_{21} = \frac{[L_2S]}{[S][L]^2} \qquad (1)$$

When $[L]_t$ is the total concentration of **1**, the concentration of probe is described as;

$$[L]_t = [L] + 2[L_2S] \qquad (2)$$

From equations (1) and (2),

$$[L] = \frac{\left(-1 + \sqrt{1 + 8K_{21}[S][L]_t}\right)}{4K_{21}[S]} \qquad (3)$$

Also, from equations (1) and (3),

$$\frac{[L_2S]}{[L]} = K_{21}[L][S] = \frac{\left(-1 + \sqrt{1 + 8K_{21}[S][L]_t}\right)}{4} \qquad (4)$$

In the case that the concentration of a fluorescence chemical species A is very low, the fluorescence intensity FI can be expressed as follows,

$$FI = \beta\phi[A] \qquad (5)$$

where ϕ is the fluorescence quantum yield and β is the device constant, respectively.

Therefore, the fluorescence ratio (FI_{dimer} / $FI_{monomer}$) can be expressed as:

$$\frac{FI_{472}}{FI_{413}} = \frac{\beta\phi_{L_{472}}[L] + \beta\phi_{(L_2S)_{472}}[L_2S]}{\beta\phi_{L_{413}}[L] + \beta\phi_{(L_2S)_{413}}[L_2S]} \qquad (6)$$

where FI_{472} and FI_{413} are fluorescence intensity of dimer (at 472 nm) and monomer (at 413 nm), respectively. Similarly, ϕ_{L413} and ϕ_{L472} are the fluorescence quantum yield for **1** at 413 and 472 nm,

$\phi_{(L2S)413}$ and $\phi_{(L2S)472}$ are the fluorescence quantum yield for the 2:1 complex at 413 and 472 nm, respectively.

From equations (5), the fluorescence ratio (FI_{dimer} / $FI_{monomer}$) can be expressed as a function of [S] and $[L]_t$, using equations (3) and (4),

$$\frac{FI_{472}}{FI_{413}} = \frac{\dfrac{\phi_{L_{472}}}{\phi_{L_{413}}} + \dfrac{\phi_{(L_2S)_{472}}}{\phi_{L_{413}}} \times \dfrac{[L_2S]}{[L]}}{1 + \dfrac{\phi_{(L_2S)_{413}}}{\phi_{L_{413}}} \times \dfrac{[L_2S]}{[L]}} = \frac{4\dfrac{\phi_{L_{472}}}{\phi_{L_{413}}} + \dfrac{\phi_{(L_2S)_{472}}}{\phi_{L_{413}}}\left(-1+\sqrt{1+8K_{21}[S][L]_t}\right)}{4 + \dfrac{\phi_{(L_2S)_{413}}}{\phi_{L_{413}}}\left(-1+\sqrt{1+8K_{21}[S][L]_t}\right)} \qquad (7)$$

In this study, $[L]_t$ is constant and the total sugar concentration $[S]_t$ is almost same as [S] (∵ $[S]_t$ >>[L]), so that equation (7) is a function of the total sugar concentration $[S]_t$. Therefore, from the equation (7), the apparent binding constant for the 2:1 inclusion complex of **1** with glucose or galactose was calculated by the least-square curve fitting analysis (KaleidaGraph^R 4.0 software; Figure S9).

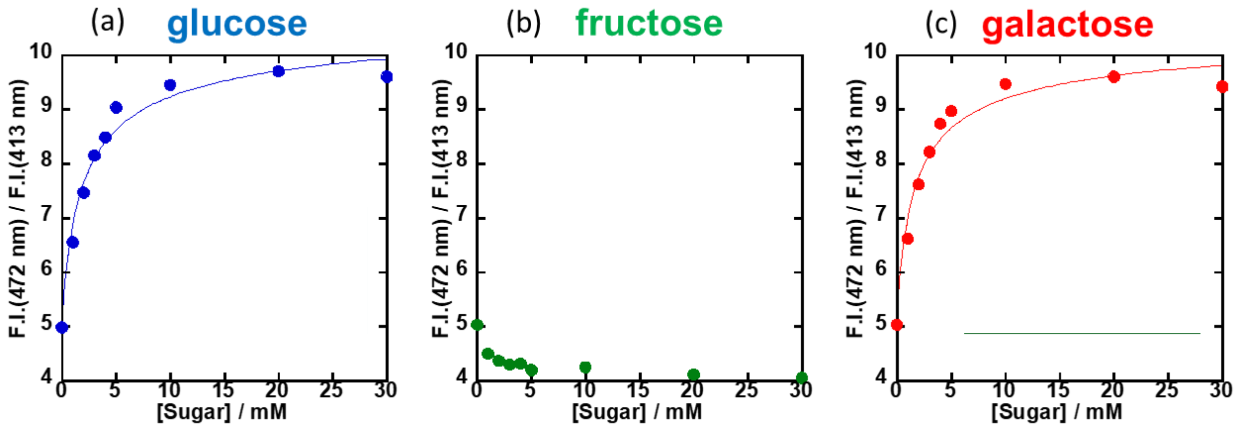

Figure S9. Ratiometric plots of **1**/γ-CyD complex in 2% DMSO - 98% water (v/v) at pH 7.4 with addition of various concentration of (a) glucose, (b) fructose and (c) galactose. [**1**] = 10 μM, [γ-CyD] = 5 mM, [sugar] = 0-30 mM, [phosphate buffer] = 10 mM, [NaCl] = 100 mM. λ_{ex} = 323 nm. The binding constant for fructose was not determined.

6. ICD spectra of 1/β-CyD complex with addition of various concentration of sugars

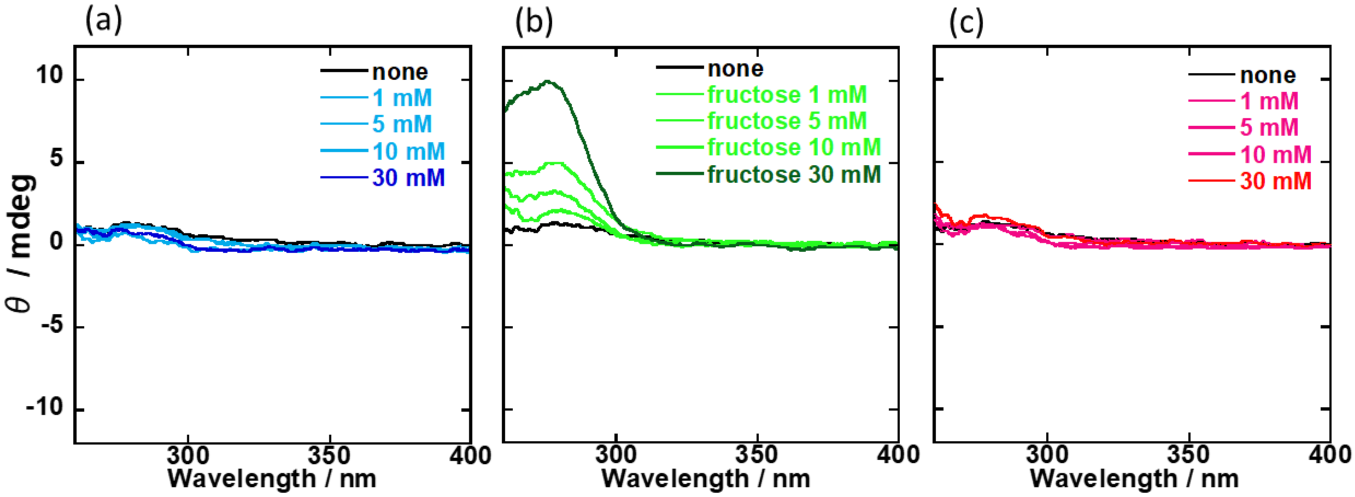

Figure S10. ICD spectra of **1**/β-CyD complex in 2% DMSO - 98% water (v/v) with addition of various concentration of (a) glucose, (b) fructose, (c) galactose at pH 7.4. [**1**] = 10 μM, [β-CyD] = 5 mM, [sugar] = 0-30 mM, [phosphate buffer] = 10 mM, [NaCl] = 100 mM.
